# Computational Mechanisms of Osmoregulation: A Reinforcement Learning Model for Sodium Appetite

**DOI:** 10.3389/fnins.2022.857009

**Published:** 2022-05-19

**Authors:** Yuuki Uchida, Takatoshi Hikida, Yuichi Yamashita

**Affiliations:** ^1^Department of Information Medicine, National Institute of Neuroscience, National Center of Neurology and Psychiatry, Tokyo, Japan; ^2^Medical and Dental Sciences, Graduate School of Medical and Dental Sciences, Tokyo Medical and Dental University, Tokyo, Japan; ^3^Laboratory for Advanced Brain Functions, Institute for Protein Research, Osaka University, Osaka, Japan

**Keywords:** computational neuroscience, decision making, homeostasis, homeostatic reinforcement learning, salt appetite

## Abstract

Homeostatic control with oral nutrient intake is a vital complex system involving the orderly interactions between the external and internal senses, behavioral control, reward learning, and decision-making. Sodium appetite is a representative system and has been intensively investigated in animal models of homeostatic systems and oral nutrient intake. However, the system-level mechanisms for regulating sodium intake behavior and homeostatic control remain unclear. In the current study, we attempted to provide a mechanistic understanding of sodium appetite behavior by using a computational model, the homeostatic reinforcement learning model, in which homeostatic behaviors are interpreted as reinforcement learning processes. Through simulation experiments, we confirmed that our homeostatic reinforcement learning model successfully reproduced homeostatic behaviors by regulating sodium appetite. These behaviors include the approach and avoidance behaviors to sodium according to the internal states of individuals. In addition, based on the assumption that the sense of taste is a predictor of changes in the internal state, the homeostatic reinforcement learning model successfully reproduced the previous paradoxical observations of the intragastric infusion test, which cannot be explained by the classical drive reduction theory. Moreover, we extended the homeostatic reinforcement learning model to multimodal data, and successfully reproduced the behavioral tests in which water and sodium appetite were mediated by each other. Finally, through an experimental simulation of chemical manipulation in a specific neural population in the brain stem, we proposed a testable hypothesis for the function of neural circuits involving sodium appetite behavior. The study results support the idea that osmoregulation *via* sodium appetitive behavior can be understood as a reinforcement learning process, and provide a mechanistic explanation for the underlying neural mechanisms of decision-making related to sodium appetite and homeostatic behavior.

## Introduction

Homeostatic systems for the control of oral nutrient intake are vital for sustaining life. These systems are quite complex, involving orderly interactions among external and internal senses, behavioral control, and reward learning (Cannon, 1929; Keramati and Gutkin, 2014). Failure to properly develop or maintain these systems with precision has been associated with several disorders, such as the homeostatic breakdown and nutrient disorders in patients with impairments in taste (Steinbach et al., 2009; Sánchez-Lara et al., 2010; Feng et al., 2014).

Sodium appetite is a representative system that has been intensively investigated in animal models of the homeostatic systems that coordinate oral nutrient intake (Richter, 1936; Watanabe et al., 2000; Tindell et al., 2009; Oka et al., 2013; Matsuda et al., 2017; Lee et al., 2019; Augustine et al., 2020). For example, at the behavioral level, it is known that preference for salty taste changes depending on the internal sodium state. Early studies revealed that, when an animal is sodium-depleted after adrenalectomy (Catalanotto and Sweeney, 1978), administration of furosemide, or low-sodium food, it exhibits a positive sodium appetite, which means that sodium intake serves as a reward. On the other hand, when an animal is not deficient in sodium, it will exhibit a negative sodium appetite, and sodium intake serves as a punishment (Galaverna et al., 1993; Chandrashekar et al., 2010). This property is not only related to avoidance of harmful foods or the consumption of essential nutrients. Rather, it is a complex phenomenon involving multiple factors, in which the reward value of a taste fluctuates, thereby reflecting the animal’s internal state.

At the physiological level, multiple hormones are involved in the control of sodium appetite. For instance, stimulation of osmoreceptors in the hypothalamus regulates release of hormones such as vasopressin and modulates the osmotic environment through kidney function (Melmed et al., 2019). Adrenalectomized rats, which have difficulty secreting aldosterone, exhibit increased sodium appetite (Richter, 1936). Sodium deficiency increases the level of angiotensin II and stimulates the secretion of aldosterone (Eaton et al., 2009). Furthermore, pacemaker-like firing of aldosterone-sensing neurons in the nucleus of the solitary tract (NTS*^HSD2^* neurons) has been observed in sodium-deficient animals (Resch et al., 2017).

Several studies have identified neural substrates involved in the control of sodium appetite, including the limbic system, pons, and basal ganglia. For example, activation of dopaminergic neurons in the ventral tegmental area (VTA), which exhibit robust correlations with reward systems, decreases salt intake (Sandhu et al., 2018). Recent evidence also indicates that dopaminergic neurons in the midbrain may encode appetitive properties of sodium (Verharen et al., 2019) and reward prediction error (Cone et al., 2016), while some excitatory neurons in the pre-locus coeruleus decrease sodium appetite (Lee et al., 2019). Conversely, the activation of neurons in the subfornical organ has been shown to increase sodium appetite (Matsuda et al., 2017).

Despite these findings, the system-level mechanisms related to the control of sodium appetite and osmoregulation remain unestablished. Researchers have offered several theoretical explanations to address this issue. For example, classical drive reduction theory (Hull, 1943) assumes that the discrepancy from the optimal state drives behavior to reduce the discrepancy, while incentive salience theory (Zhang et al., 2009; Berridge, 2012) assumes that the “incentive” to consume sodium switches depending on the internal sodium state. However, some aspects of these theories have not been adequately considered or explained, such as the effects of taste (Lee et al., 2019) and multiple drives related to water and sodium (Matsuda et al., 2017). In the current study, we developed a computational homeostatic reinforcement learning (HRL) model to investigate the mechanistic control of sodium appetite.

As an evolution of drive reduction theory, the HRL model interprets homeostatic behaviors as reinforcement learning processes (Keramati and Gutkin, 2014; Keramati et al., 2017; Hulme et al., 2019). In the HRL model, reductions of drive (physiological needs) are regarded as rewards, while increases are regarded as punishments. Based on this idea, the values of the optimal behavior for maintaining internal states are acquired through an incremental learning process. In addition, by treating the taste modality and the actual change in the internal state separately, the HRL model provides explanations regarding the mechanisms that integrate taste, behavior, and the maintenance of the internal environment (Keramati and Gutkin, 2014; Keramati et al., 2017; Hulme et al., 2019; Petzschner et al., 2021).

The HRL model was originally proposed to explain the homeostatic control of body temperature, internal water balance (Keramati and Gutkin, 2014), and pathological mechanisms related to cocaine addiction (Keramati et al., 2017). This study is the first attempt to use the HRL model to explain sodium appetite behavior. In addition, although the HRL model can handle multi-dimensional internal states, previous studies have utilized it to examine one-dimensional changes in internal states (Keramati and Gutkin, 2014; Keramati et al., 2017; Hulme et al., 2019). However, sodium appetite is a complex process that involves interactions between the homeostatic balance and the preferences for water and sodium. Therefore, in the current study, we introduced a multi-dimensional version of the HRL model incorporating the internal states of both water and sodium balance, to provide a mechanistic understanding of previous findings. Finally, in our simulation experiment, we manipulated neural activity in a particular brain nucleus known to be involved in sodium appetitive behavior, allowing us to provide a hypothesis regarding the role of this population in the control of sodium appetite.

## Materials and Methods

### Sodium Homeostasis

In the current study, sodium appetitive behavior was modeled using the HRL model. This model is based on the assumption that homeostasis is an RL process, in which the minimization of deviations in internal states from an optimal level (i.e., homeostasis) is treated as a computation for maximizing the sum of rewards. In the HRL model, a multi-dimensional metric space in which each dimension represents an internal state (such as body temperature, blood glucose density, water balance, and sodium level) is defined as the “homeostatic space.” In this homeostatic space, the drive function *D(H_*t*_)* is defined as the distance between the internal state of the *i*-th component (e.g., water or sodium) at time *t*, *H*^i^*_*t*_*, and the ideal internal state *H*^^*i^:


(1) $$\begin{array}{c} D\,\left(H_{t}\right)=\sqrt[{m}]{\sum_{i=1}^{N}{\left|H^{*i}-H_{t}^{i}\right|}^{n}} \end{array}$$

where *m* and *n* are free parameters that define the distance, and *N* is the total number of dimensions for internal states (e.g., water, sodium, etc.). When the internal state approaches the ideal state, the value of the drive function should be reduced. Based on this drive function, the reward *r*_*t*_ is determined as a change in the values of the drive function from time *t* to time *t* + *1*. Specifically, to implement nutrient intake, the internal state at time *t* + *1* should contain the amount of nutrient intake at time *t*, defined as *K*_*t*_:


(2) $$r\,\left(H_{t},K_{t}\right)=D\,\left(H_{t}\right)-D\,\left(H_{t+1}\right)\,\begin{array}{c} =D\,\left(H_{t}\right)-D\,\left(H_{t}+K_{t}\right) \end{array}$$

As described later, in the HRL model, the intake of taste stimuli (${\hat{K}}_{t}$) can be modeled as a predictor of the actual nutrient intake (*K*_*t*_). Under this assumption, the reward was calculated as follows:


(3) $$\begin{array}{c} r\,\left(H_{t},{\hat{K}}_{t}\right)=D\,\left(H_{t}\right)-D\,\left(H_{t}+{\hat{K}}_{t}\right) \end{array}$$

**FIGURE 1:**
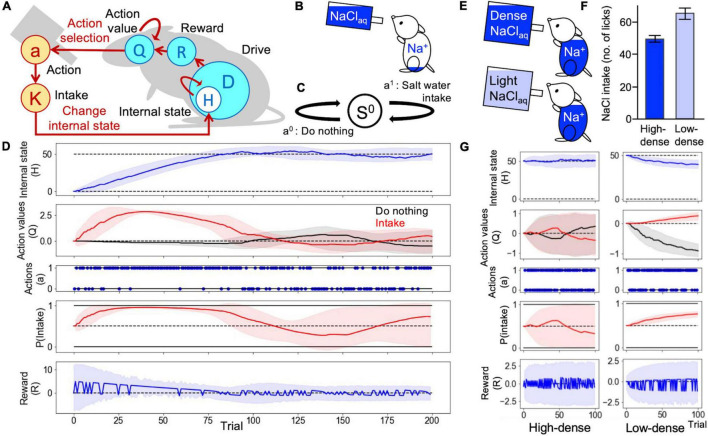
Homeostatic behavior according to the homeostatic reinforcement learning (HRL) model. **(A)** Schematic drawing of the computational process of the HRL model. **(B)** In an assumed animal behavior, sodium-depleted mice were able to lick saltwater. **(C)** Definition of a state and two actions in Simulations 1 and 2. **(D)** Example of homeostatic behavior. Changes in internal sodium state (*H*), the value of each action (*Q*-value), selected actions (*a*), probability of sodium intake [*P*(*Intake*)], and magnitude of reward (*R*) over time are plotted. The solid lines represent the results of a trial, and the light-colored error ranges represent the mean ± 2 SD of 100 trials. The dotted line in the panel of the internal state indicates the ideal point (*H*^^*^ = 50) of sodium taste. In the panel related to actions (*a*), action 1 represents “Intake behavior,” and action 0 indicates “do nothing.” At the beginning of the simulation, the internal sodium state and *Q*-values for each action were set to 0. After several random selections of action, the *Q*-value of sodium intake was increased, and the internal sodium state quickly reached the ideal point, maintaining homeostatic regulation of behavior. **(E)** In assumed animal behaviors, a group of sodium-repleted mice was able to lick high-density saltwater, and the other licked low-density. **(F)** The number of licks of high-density saltwater was fewer than that of low-density. **(G)** Transitions of each variable over time.


The Rescorla–Wagner model was used to model the RL process. In this model, the values of action *a*_*t*_ (e.g., sodium intake, do nothing…) and *Q_*t*_(a)* are updated based on the reward prediction error:


(4) $$\begin{array}{c} Q_{t+1}\,\left(a\right)=Q_{t}\,\left(a\right)+\alpha^{Q}\,\left(r_{t}-Q_{t}\,\left(a\right)\right) \end{array}$$

where α*^Q^* is the learning rate for *Q_*t*_(a)*. To investigate the applicability of the HRL model to sodium appetite behavior, we performed a sodium intake test (Simulation 1). The computation algorithm is illustrated in Figure 1A. In this simulation, only the internal state of sodium was considered. An external state (*S*^0^) and two actions, do nothing (*a*^0^) and intake (*a*^1^), were assessed (Figures 1B,C). Action selection depends on the relative magnitudes of the values of each action (*Q*-value), following the soft-max function:

(5) $$\begin{array}{c} P_{t}\,\left(a_{t}^{k}\right)=\frac{e\,x\,p\,\left(\beta \,\text{⋅}\,Q_{t}\,\left(a_{t}^{k}\right)\right)}{\sum_{j}e\,x\,p\,\left(\beta \,\text{⋅}\,Q_{t}\,\left(a_{t}^{j}\right)\right)} \end{array}$$

where *P_*t*_(a*^k^*)* is the probability of an action *a^k^* to be selected at time *t*, and β is the inverse temperature, a parameter controlling the randomness of an action. In Simulation 1, the *Q*-values of both actions were set to 0. Therefore, the first action was randomly chosen. When intake behavior was performed, the internal sodium state increased with *K*_*t*_, a constant parameter defining the amount of sodium intake. When nothing was chosen, *K*_*t*_ was set to 0. At *t* = 0, to represent sodium depletion, the first internal state (*H*_0_ = 0) was far lower than the ideal state (H* = 50). At this stage, the value of the drive function was large because the drive function corresponds to a type of distance from the internal state of time *t* (*H*_*t*_) to the ideal state (H* = 50) (Equation 1). If an agent performed the intake behavior at this moment, the internal state increased and the drive function became smaller, resulting in a positive reward (Equation 2).

In addition, the natural decrease in sodium balance was implemented as follows using the temporal decay constant τ (Equation 6).


(6) $$\begin{array}{c} H_{t+1}=\left(1-\frac{1}{\tau }\right)\,H_{t} \end{array}$$

As a result, the calculation of the reward value was determined as follows (Equation 7):


(7) $$r\,\left(H_{t},K_{t}\right)=D\,\left(H_{t}\right)-D\,\left(H_{t+1}\right)\,\begin{array}{c} =D\,\left(H_{t}\right)-D\,\left(\left(1-\frac{1}{\tau }\right)\,H_{t}+K_{t}\right) \end{array}$$

To update *Q*-values based on the reward value, we used a conventional Rescorla–Wagner model (Sutton and Barto, 2018), where *i* indicates each action, α*^Q^* is the learning rate, and *r*_*t*_—*Q_*t*_(a)* represents the reward prediction error. After this update of the *Q*-values, the agent chooses the next action. The detailed values of the simulation parameters are listed in Supplementary Table 1.

As mentioned, the HRL theory assumes that ${\hat{K}}_{t}^{a}$, the cognition of the stimulus based on the reward from the action *a*_*t*_, is renewed through learning. In the present study, the following equation was used to update ${\hat{K}}_{t}^{a}$:


(8) $$\begin{array}{c} {\hat{K}}_{t+1}^{a}={\hat{K}}_{t}^{a}+\alpha^{\hat{K}}\,\left(K_{t}^{a}-{\hat{K}}_{t}^{a}\right) \end{array}$$

In addition, concentration of sodium was defined as the amount of nutrient intake at time *t* (defined as *K*_*t*_), namely, a smaller value of *K*_*t*_ for low-density saltwater. Concrete parameters used in the simulations are represented in Supplementary Table 1.

### Oral Sense as a Predictor of Changes in Internal States

In the previous HRL model, the sense of taste was hypothesized to predict changes in the internal state. As such, we introduced oral sense ${\hat{K}}_{t}$, which represents the prediction of changes in the internal state, in Simulations 2–4. The definition of the reward (Equation 3) was also updated as follows:


(9) $$\begin{array}{c} r\,\left(H_{t},\hat{K_{t}}\right)=D\,\left(H_{t}\right)-D\,\left(\left(1-\frac{1}{\tau }\right)\,H_{t}+{\hat{K}}_{t}\right) \end{array}$$

Thus, the reward was defined as changes in internal states, and the taste input was used as a predictor for changes in internal states. The other functions were the same as those in Simulation 1.

**FIGURE 2:**
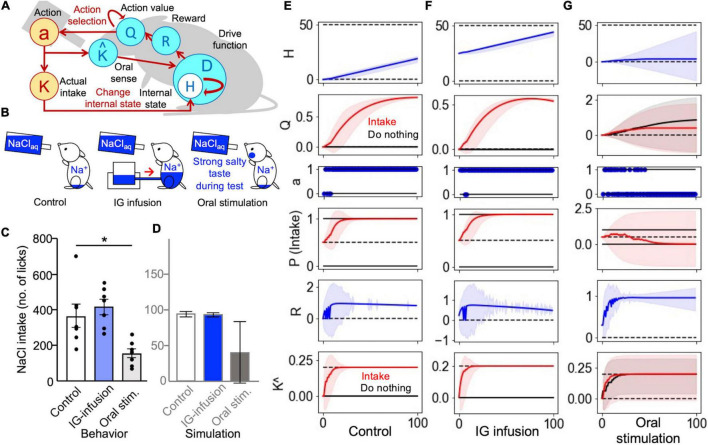
Intragastric and oral stimulation tests were explained using the sense of taste as a predictor of changes in the internal state. **(A)** Schematic illustration of Simulations 2 and 3: Taste perception (*K*^) was a predictor of an increase in the internal state. **(B)** Three groups for the behavioral tests. **(C)** The results of the behavioral experiment (Lee et al., 2019). *P < 0.05. **(D)** The results of the computational model. Intragastric infusion did not change the level of sodium intake, while oral stimulation decreased intake. **(E–G)** Transitions of the simulation. **(E)** The control group exhibited an increased *Q*-value for intake. **(F)** A congenial increase in the *Q*-value was observed in the IG-infusion group. **(G)** The values of “do nothing” and “intake” were reinforced in the oral-stimulation group. **(E–G)** The solid lines represent the results of a trial, and the light-colored error ranges represent the mean ± 2 SD of 100 simulated agents.

The details of the behavioral experiment used to investigate intragastric infusion are shown in Figures 2A,B. The animals were separated into three groups: control, intragastric, and oral stimulation. Control animals underwent sodium depletion only. In addition to sodium depletion, animals in the intragastric (IG) infusion group underwent insertion of an intragastric cannula into the gut, through which saltwater was directly infused prior to the intake test. In the oral stimulation group, the animals were stimulated with a strong salty stimulus during the intake test. To model the IG-infusion group, we hypothesized that the internal state of sodium was set to the level of half-satisfaction at the beginning of the intake test. To model the oral stimulation group, a salty taste was supplied, regardless of the selected actions. The definitions of states and actions were the same as in Simulation 1 (Figure 1C). The algorithm including taste input as a predictor of changes in the internal state is illustrated in Figure 2A. The detailed values of the parameters used in this simulation are shown in Supplementary Table 2.

### Two-Bottle Preference Test

For the simulation of the two-bottle preference test in Simulation 3, we set two internal states corresponding to water and sodium states. Thus, in Equation 1, the number of dimensions of internal state *N* was set to 2. Each internal state updates as follows:


(10) $$\begin{array}{c} H_{t+1}^{i}=\left(1-\frac{1}{\tau^{i}}\right)\,H_{t}^{i} \end{array}$$

where *i* represents each dimension of internal state, i.e., water or sodium.

The detailed parameters were partially different from those of Simulation 2. The detailed values of the parameters used for Simulation 3 are shown in Supplementary Table 3.

### Designer Receptors Exclusively Activated by Designer Drugs Experiment

In the simulated designer receptors exclusively activated by designer drugs (DREADD) experiment, we assumed that LPBN*^Htr2c^* neurons provided tonic suppression of sodium appetite as implemented by a tonic negative bias in the selection of sodium intake action. That is, the LPBN-amygdala projection provides a negative bias of action selections and has no direct contribution to the learning of the *Q*-value, as follows:


(11) C⁢o⁢n⁢t⁢r⁢o⁢l:Qs′=Qs-L⁢P⁢B⁢N

where *Q^s^* is *Q*-value for saltwater intake, and *LPBN* is a positive constant value corresponding to the negative bias of the LPBN*^Htr2c^* neuron. Note that *Q’* is used only for action selections, and *Q*-values are updated with previous *Q*-values and the reward, regardless of *Q’* and the tonic bias (Equation 11).

DREADD treatment was implemented as the cancelation of this negative bias by adding the positive value of *drd* as follows:


(12) D⁢R⁢E⁢A⁢D⁢D:Qs′=Qs-L⁢P⁢B⁢N+d⁢r⁢d

In the current simulation, the values of *LPBN* and *drd* were set to be the same. At the beginning of the saltwater intake test, the initial *Q*-value for salt intake behavior was set to *LPBN*, corresponding to the state, where *Q’* for both actions was 0 (i.e., the probabilities for salt intake and do-nothing were 0.5). The initial water state was set to 0 for the dehydration group, and the initial sodium state was set to 0 for the sodium-depleted group. The detailed values of the parameters for Simulation 3 are shown in Supplementary Table 4.

## Results

### Simulation 1: Sodium Homeostasis According to the Homeostatic Reinforcement Learning Model

First, we confirmed that the homeostatic control of the internal sodium state can be replicated using the framework of the HRL model. In this simulation, mice were able to choose to either perform saltwater intake or do nothing (Figures 1B,C). The action values of intake and do nothing were both set to 0. Therefore, the initial action selection was random [*P(intake)* = 0.5] (Figure 1D). After several random choices of sodium intake, the action value of intake was reinforced, and the internal state approached the ideal state. After approximately 20 trials, *P(intake)* was nearly 1, and the internal state rapidly reached the ideal state. When the internal state exceeded the ideal state after approximately 80 trials, sodium intake became a punishment, and the action value of do nothing increased. After several trials of do nothing, at around trial number 140, due to the decay assumed to be a natural loss of internal sodium (see section “Materials and Methods” for more details), the internal state became lower than the ideal state. Therefore, the action value of sodium intake increased again. Through repetitions of this cycle, the model successfully achieved homeostatic control of the internal sodium state (Figure 1D). Additionally, we tested the dose-dependent changes in preference to saltwater in HRL mode, namely repleted mice preferred low-density saltwater (∼ 100 mM NaCl) (Oka et al., 2013). In this simulation with the state definition the same as in Figure 1C, two groups of subjects were set: one can lick high-density saltwater and the other can lick low-density saltwater, represented with a small amount of sodium in an intake (Figure 1E). As a result, the low-density group showed high preference toward saltwater (Figures 1F,G) consistent with the biological observation.

### Simulation 2: Sense of Taste as a Predictor of Changes in Internal States

The sense of taste may play an important role in the homeostatic control of sodium balance and in the monitoring of internal states. In the HRL model, this assumption can be tested by implementing taste as a predictor of changes in the internal states induced by nutrient intake (Keramati and Gutkin, 2014). In this study, we simulated an intragastric infusion test (Lee et al., 2019) using three groups of animals: (1) a control group of sodium-depleted mice, (2) an IG-infusion group of sodium-depleted animals treated with an intragastric infusion of saltwater before the test, and (3) an oral-stimulation group of sodium-depleted mice stimulated with sodium (salty taste) during the test (Figure 2B). The definitions of the states and actions were the same as in the previous simulation (Figure 1C). At the beginning of the intragastric infusion test, the internal states for the control group and oral-stimulation group were set to *H*_*t*_ = 0, while it was set to *H_*t*_* = H*/2 for the IG-infusion group, corresponding to sodium partially supplied through a gastric infusion. Each animal model was tested in 100 trials, with a total duration of 600 s in the actual experiments. During the 100 trials, the model animals of the oral-stimulation group were assumed to have constant salty taste stimulation (Figure 2B).

In this simulation, there were no significant differences in the total number of NaCl intake steps between the control and IG-infusion groups (Figure 2D). However, the total intake of the oral-stimulation group was clearly lower than that of the control and IG-infusion groups. These trends were similar to those observed in animal experiments (Lee et al., 2019; Figure 2C).

To provide a mechanistic overview of these results, the transitions of each variable during the simulation are plotted in Figures 2E–G. In the IG-infusion group, even though sodium was partly supplied through gastric infusion, an increase in the action value of sodium intake led to an increase in sodium intake behavior (Figure 2F), resulting in the number of intakes not changing significantly when compared with the control group (Figure 2D). In the oral-stimulation group, the action value of do nothing was reinforced (Figure 2G) based on the expectation of an increase in the internal sodium state (${\hat{K}}_{t}$) due to the continuous application of the salty stimulus (sodium) during the test. There were no clear differences between the action values of salt intake and do nothing (Figure 2G), and the number of intakes of the group that received NaCl as drinking water was lower than that of the control group (Figure 2D).

### Simulation 3: Multi-Dimensional Homeostatic Reinforcement Learning

**FIGURE 3:**
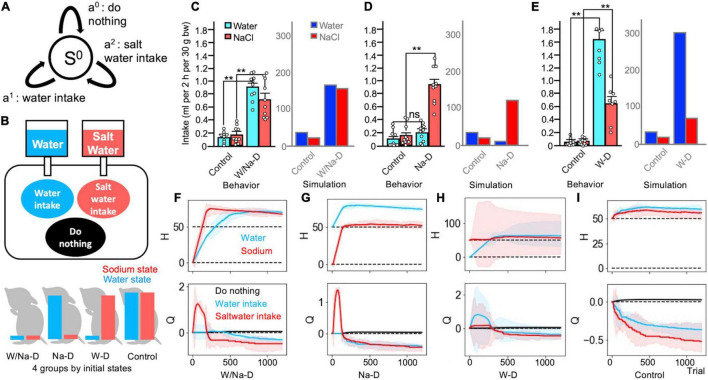
Multi-dimensional homeostatic reinforcement learning (HRL) model as a suitable explanation of findings in the two-bottle preference test. **(A)** Definition of a state and three actions in Simulations 3 and 4. **(B)** Schematic drawing of Simulation 3 (two-bottle preference test). **(C)** Comparison of behavioral data (Matsuda et al., 2017) and simulated data in the control (all-satisfied) and water/sodium-depleted groups. Control groups exhibited minor intake, and both depleted groups demonstrated copious volumes of intake in both sets of data. **(C–E)** Data averaged over 100 simulated agents. **P < 0.01. **(D)** In the sodium-depleted group, water intake was slight, while saltwater intake was increased. **(E)** The water-deficient groups exhibited abundant water intake and non-negligible saltwater intake. **(F–I)** Transitions of the HRL models. **(F)** The water/sodium-deficient HRL model exhibited strong increases in the values of water and saltwater intake. **(G)** The action value for sodium intake soared in the sodium-depleted model. **(H)** The water-depleted model exhibited increased values for water and saltwater intake. **(I)** The control model refused both intakes. **(F–I)** The solid lines represent the results of a trial, and the light-colored error ranges represent the mean ± 2 SD of 100 simulated agents.

In this simulation, in order to describe the dynamic interaction between the internal states of water and sodium, we extended the HRL model to multiple dimensions based on the idea proposed in a previous study (Keramati and Gutkin, 2014). The HRL model with multi-dimensional internal states (water and sodium states) was assessed *via* a two-bottle preference test, which is a behavioral procedure used to compare the preference toward the contents of two bottles (Figure 3B). In the two-bottle preference test, the model mice were able to either perform “saltwater intake,” “water intake,” or “do nothing” (Figure 3A). In this experiment, there were four groups of animals: a control group with initially fulfilled internal states, a sodium-depleted group, a water-depleted group, and a water/salt-depleted group.

The results of the simulations revealed that the control group with the initially fulfilled internal states exhibited continuously decreased action values of both water intake and saltwater intake, and the individuals in this group mostly chose to perform the do-nothing behavior. As a result, both internal states remained flat in the ideal state (Figure 3I). Accumulating these intakes, consumption from water bottles and saltwater bottles was low-keyed (Figures 3C–E). In contrast, in the water/salt-depleted group, the action values of both water intake and saltwater intake rapidly increased. Reflecting these increases, both the water and sodium states also increased (Figure 3F). The accumulation of these consumptions was evidently larger in the depleted group than in the control group (Figure 3C). In other circumstances, the sodium-depleted group, which was provided with large amounts of water, exhibited an increased action value for saltwater intake, resulting in an increased sodium state (Figure 3G). The total intake of water was slight, whereas saltwater intake was dominant (Figure 3D). The water-deficient group exhibited notable behaviors. First, both the action values of water intake and salt intake increased, although the action value of water intake was larger than that of saltwater intake. Saltwater intake slightly increased (Figure 3H). These trends were similar to those observed in the actual animals assessed using the two-bottle preference test (Matsuda et al., 2017; Figure 3C).

### Simulation 4: Simulated Chemogenetic Neural Manipulation in the Sodium Appetite Network

**FIGURE 4:**
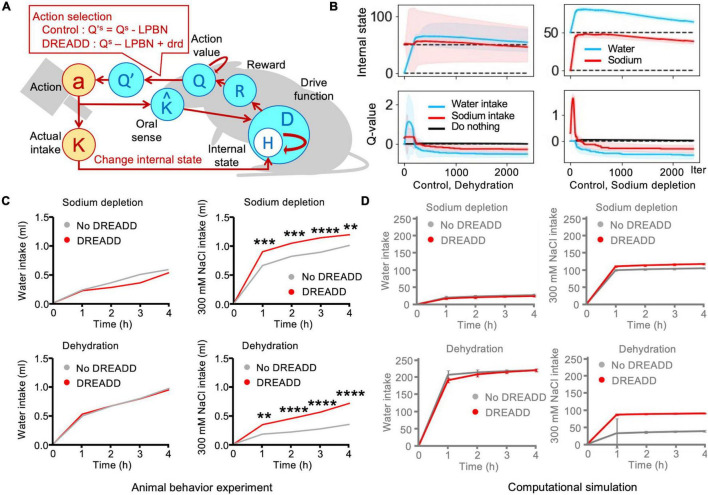
Simulation of designer receptors exclusively activated by designer drugs (DREADD), which involved tonic neuronal suppression of sodium appetite. **(A)** Schematic illustration of Simulation 4: DREADD. **(B)** Transitions in the control (no-DREADD) models. Sodium intake values were subtracted from these plots by a negative bias toward sodium appetite. The solid lines represent the results of a trial, and the light-colored error ranges represent the mean ± 2 SD of 100 simulated agents. **(C)** DREADD experiments increased sodium intake (Park et al., 2020). **P < 0.01, ***P < 0.001, and ****P < 0.0001. **(D)** Homeostatic reinforcement models demonstrated equivalent behaviors. The data are averaged over 100 simulated agents.

Neurons in the lateral parabrachial nucleus (LPBN*^Htr2c^* neurons) are assumed to play a role in suppressing sodium appetite, as previous studies have indicated that artificial inhibition of these neurons *via* chemogenetic neural manipulation (e.g., DREADD) increases sodium appetite (Park et al., 2020). LPBN*^Htr2c^* neurons project to the central amygdala (CeA). Based on these previous findings, in this simulation, we hypothesized that LPBN*^Htr2c^* neurons inflict a negative bias on the action value of sodium appetitive behavior. The hypothesis was implemented as an account of action values in the HRL model, as Equation 11. Additionally, the inhibition of LPBN*^Htr2c^* neurons by DREADD was represented by the cancelation of the negative bias (i.e., *LPBN* in Equation 11 was set to 0). With this implementation, we tested our hypothesis regarding LPBN neurons by comparing the simulation with the results observed in an actual animal experiment (Figure 4A; Park et al., 2020).

This experiment involved a two-bottle preference test using water and saltwater (Park et al., 2020). There were four groups of mice based on the depletion of water/salt and application of DREADD, namely water-depleted/control (no DREADD), sodium-depleted/control, water-depleted/DREADD, and sodium-depleted/DREADD. Both DREADD groups exhibited greater intake of saltwater than the control group. However, no clear differences were observed between the no-DREADD groups. Among sodium-depleted animals, water intake was slight in both the no-DREADD and DREADD groups. In both groups, saltwater intake rapidly increased, although the intake of the DREADD group was higher than that of the no-DREADD group. Water intake increased sharply in the dehydration groups. There was no clear difference between the DREADD and no-DREADD groups. The control (no-DREADD) model exhibited maintenance of homeostasis (Figure 4B). Saltwater intake was slight in the dehydration groups, although intake was much higher in the DREADD group than in the no-DREADD group. These trends successfully replicated those observed in the actual animal experiments (Figures 4C,D).

## Discussion

In this study, we attempted to provide a mechanistic understanding of sodium appetite behavior using the HRL model. In Simulation 1, we confirmed that the HRL model successfully reproduced homeostasis-like behaviors by regulating sodium appetite in concentration-depending manner, (i.e., approach and avoidance behavior to sodium). In addition, based on the assumption that the sense of taste is a predictor of changes in internal states, the HRL model successfully reproduced the previous observations of the intragastric infusion test that cannot be explained by classical drive reduction theory (Hull, 1943). These results support the idea that sodium appetitive behavior can be understood as an RL process.

This idea is consistent with previous findings that the reward learning system is involved in sodium appetite behaviors. For instance, the activity of dopaminergic neurons in the VTA, which is thought to exhibit a robust relationship with the RL process in the brain (Nakanishi et al., 2014; Schultz, 2015), increases when sodium-depleted mice lick saltwater. In contrast, pharmacological inactivation of neural projections from the VTA to the nucleus accumbens decreases sodium intake (Verharen et al., 2019). In addition, recent studies have indicated that optogenetic excitation of VTA dopaminergic neurons suppresses sodium intake in sodium-depleted mice (Sandhu et al., 2018). As described later, the HRL model may aid in integrating these previous findings.

However, there also exists a theoretical model explaining changes in sodium appetite from a different perspective. Incentive salience theory argues that the incentive for sodium is determined based on the animal’s internal states and is naturally independent from the learning process (Zhang et al., 2009; Berridge, 2012). Indeed, this model successfully reproduces not only approach and avoidance behavior to sodium, but also explains the puzzling observation that a negatively conditioned stimulus can be immediately switched to a preferred stimulus without learning (Zhang et al., 2009; Berridge, 2012). In the HRL model, switching of preference takes some time due to the involvement of the learning process. Therefore, an additional mechanism may be necessary for the HRL to integrate this aspect of sodium appetite. We discuss this point in the later section.

In the HRL model, the sense of taste was hypothesized to predict changes in the internal state. The latter means that the salty stimulus represents an immediate inducer of reinforcement, but this was not the case for the actual changes in the internal state. Nevertheless, only actual intake may result in the satiation of internal states. This assumption is consistent with the fact that gastric infusion of water does not act as a reinforcer, in contrast to oral intake of water, which can indeed act as a reinforcer (McFarland, 1969; Keramati and Gutkin, 2014). In addition, artificial sweeteners, including saccharine and sucralose, can function as reinforcers (Hughes, 1957; Collier and Siskel, 1959; Fernandes et al., 2020), although their effects are relatively weaker than those of sucrose, which induces substantial changes in the internal state. From the perspective of computational theory, this assumption of the HRL model corresponds to predictive processing (also referred to as predictive coding or active inference) theory, in the sense that homeostasis is understood as the prediction of interoceptive sensory states and minimization of prediction error (Friston, 2010). As such, homeostasis and sodium appetite behavior may provide an ideal research setting for unifying these computational theories.

Therefore, in Simulation 3, we extended the HRL model to multi-modal data, successfully reproducing the results of behavioral tests in which water and sodium appetite regulated one another. As the simplest attempt of the current study, the internal states of sodium and water were assumed to contribute equally. However, in an actual biological system, the homeostatic maintenance of water and sodium may not be exactly equal. As described later, the effects of an intragastric infusion of water and saltwater on the respective appetite for each may occur over different timescales (Matsuda et al., 2017; Augustine et al., 2020). A more detailed implementation of such differences in water and sodium appetite may provide novel insights for understanding the system-level mechanisms underlying sodium appetite.

In Simulation 4, we successfully replicated the characteristic features of LPBN*^Htr2c^* suppression experiments using DREADD. In the proposed model, the LPBN-amygdala projection provides a negative bias of action selections and has no direct contribution to the learning of the *Q*-value. This assumption is consistent with previous findings that an immediate increase in sodium craving *via* sodium depletion may not be mediated by learning processes (Tindell et al., 2006). As such, this assumption of the tonic negative bias toward sodium appetite may help to integrate the incentive salience model into the HRL.

In Simulation 2, the oral-stimulation group had large behavioral variations. This is because in the oral-stimulation group, strong salty taste was given not only during saltwater intake, but also during do-nothing, with the result that the behavioral value of intake and do nothing was less pronounced. As such, the choice of actions was more likely to be varied (large variation). This observation in the simulation is consistent with a previous animal experiment (Lee et al., 2019), in which the range of error appeared to be large in the NaCl-oral group.

In addition, this assumption is consistent with the previous findings in the sense that the CeA is an essential region for both hedonic and aversive intakes. For example, CeA pre-pronociceptin-expressing neurons are activated by hedonic intake and promote palatable food consumption (Hardaway et al., 2019). In contrast, activation of *PKC-*δ + neurons in the lateral subdivision of the CeA inhibits feeding (Cai et al., 2014). Further investigation of the neural connections from the LPBN to the CeA, together with the HRL model, may provide fundamental information for the development of more precise algorithms.

Here, we discuss the significance of constructing a computational model for sodium appetite. To understand complex systems such as the brain, investigations from three levels are essential, namely computational theory, representations and algorithms, and hardware implementation (Marr, 2010). In this study, we provided a mechanistic explanation of sodium appetite behavior by bridging previous findings related to these three levels. Although the model behaviors were evaluated only for their quantitative similarities with the actual animal experiments, the model can also provide quantitative predictions of unobservable latent variables, such as reward prediction error, action values (motivation toward nutrient intake), and predicted internal states. Investigating the neural correlates of such latent variables may provide a deeper understanding of the neural mechanisms underlying sodium appetite and homeostatic behavior. For example, as reported in Cone et al. (2016), reward prediction error of sodium appetite corresponds to dopaminergic activity. Incorporating these findings into the HRL model may be among the promising directions for future research.

Notably, the current study had several other limitations. For example, the *m* and *n* parameters in Equation 1 which control the shape of homeostatic space were transferred from Keramati and Gutkin (2014), but were not fully investigated in this study. Regarding the assumptions for chemical acts, Simulation 4 assumed that the DREADD manipulation perfectly deactivated the target neuron, i.e., ignored the degree of inhibition, effects of clozapine *N-*oxide (CNO) metabolites, and backpropagation effects. In addition, the implementation of “internal state” in the current model was not sufficient to represent diverse time constants, i.e., it could not represent different timescales for taste, gut, blood concentration, etc. (Ichiki et al., 2022). Moreover, although this study only replicated existing animal studies, it would be useful to propose a working hypothesis for actual animal experiments, for example, by using the combination of operant conditioning learning tasks and optogenetics method. Finally, osmotic homeostasis seems to have a higher priority than the homeostasis of water and sodium (Bourque, 2008). However, such hierarchy is beyond the scope of the current study. To implement this ranking relationship, future studies may wish to construct each homeostatic process in a hierarchical manner (e.g., active inference model or the free energy principle) (Pezzulo et al., 2015; Stephan et al., 2016).

## Data Availability Statement

The datasets presented in this study can be found in https://github.com/YuukiUchida/Uchida2022_OsmoHRL.

## Author Contributions

YU conceived the study, performed the experiments, and analyzed the data. YU, TH, and YY designed the experiments, performed the analyses, and wrote the manuscript. All authors contributed to the article and approved the submitted version.

## Conflict of Interest

The authors declare that the research was conducted in the absence of any commercial or financial relationships that could be construed as a potential conflict of interest.

## Publisher’s Note

All claims expressed in this article are solely those of the authors and do not necessarily represent those of their affiliated organizations, or those of the publisher, the editors and the reviewers. Any product that may be evaluated in this article, or claim that may be made by its manufacturer, is not guaranteed or endorsed by the publisher.

## References

[B1] Augustine V. Lee S. Oka Y. (2020). Neural control and modulation of thirst, sodium appetite, and hunger. *Cell* 180 25–32. 10.1016/j.cell.2019.11.040 31923398 PMC7406138

[B2] Berridge K. C. (2012). From prediction error to incentive salience: mesolimbic computation of reward motivation. *Eur. J. Neurosci.* 35 1124–1143. 10.1111/j.1460-9568.2012.07990.x 22487042 PMC3325516

[B3] Bourque C. W. (2008). Central mechanisms of osmosensation and systemic osmoregulation. *Nat. Rev. Neurosci.* 9 519–531. 10.1038/nrn2400 18509340

[B4] Cai H. Haubensak W. Anthony T. E. Anderson D. J. (2014). Central amygdala PKC-δ+ neurons mediate the influence of multiple anorexigenic signals. *Nat. Neurosci.* 17 1240–1248. 10.1038/nn.3767 25064852 PMC4146747

[B5] Cannon W. B. (1929). Organization for physiological homeostasis. *Physiol. Rev.* 9 399–431. 10.1152/physrev.1929.9.3.399

[B6] Catalanotto F. A. Sweeney E. A. (1978). Salivary sodium and potassium concentrations in adrenalectomized rats. *Behav. Biol.* 24 467–473. 10.1016/S0091-6773(78)90803-9 747584

[B7] Chandrashekar J. Kuhn C. Oka Y. Yarmolinsky D. A. Hummler E. Ryba N. J. P. (2010). The cells and peripheral representation of sodium taste in mice. *Nature* 464 297–301. 10.1038/nature08783 20107438 PMC2849629

[B8] Collier G. Siskel M. (1959). Performance as a joint function of amount of reinforcement and inter-reinforcement interval. *J. Exp. Psychol.* 57 115–120. 10.1037/h0040857 13641582

[B9] Cone J. J. Fortin S. M. McHenry J. A. Stuber G. D. McCutcheon J. E. Roitman M. F. (2016). Physiological state gates acquisition and expression of mesolimbic reward prediction signals. *Proc. Natl. Acad. Sci. U.S.A.* 113, 1943–1948. 10.1073/pnas.151964311 26831116 PMC4763767

[B10] Eaton D. C. Pooler J. Vander A. J. (2009). *Vander’s Renal Physiology.* New York: McGraw-Hill Medical.

[B11] Feng P. Huang L. Wang H. (2014). Taste bud homeostasis in health, disease, and aging. *Chem. Sens.* 39 3–16. 10.1093/chemse/bjt059 24287552 PMC3864165

[B12] Fernandes A. B. Alves da Silva J. Almeida J. Cui G. Gerfen C. R. Costa R. M. (2020). Postingestive modulation of food seeking depends on vagus-mediated dopamine neuron activity. *Neuron* 106 778.e–788.e. 10.1016/j.neuron.2020.03.009 32259476 PMC7710496

[B13] Friston K. (2010). The free-energy principle: A unified brain theory? *Nat. Rev. Neurosci.* 11 127–138. 10.1038/nrn2787 20068583

[B14] Galaverna O. G. Seeley R. J. Berridge K. C. Grill H. J. Epstein A. N. Schulkin J. (1993). Lesions of the central nucleus of the amygdala I: effects on taste reactivity, taste aversion learning and sodium appetite. *Behav. Brain Res.* 59 11–17. 10.1016/0166-4328(93)90146-H 8155277

[B15] Hardaway J. A. Halladay L. R. Mazzone C. M. Pati D. Bloodgood D. W. Kim M. (2019). Central amygdala Prepronociceptin-expressing neurons mediate palatable food consumption and reward. *Neuron* 102 1037.e–1052.e. 10.1016/j.neuron.2019.03.037 31029403 PMC6750705

[B16] Hughes L. H. (1957). Saccharine reinforcement in a T maze. *J. Comp. Physiol. Psychol.* 50 431–435. 10.1037/h0044372 13481177

[B17] Hull C. L. (1943). *Principles of Behavior: An Introduction to Behavior Theory.* New York: Appleton-Century.

[B18] Hulme O. J. Morville T. Gutkin B. (2019). Neurocomputational theories of homeostatic control. *Phys. Life Rev.* 31 214–232. 10.1016/j.plrev.2019.07.005 31395433

[B19] Ichiki T. Wang T. Kennedy A. Pool A. H. Ebisu H. Anderson D. J. (2022). Sensory representation and detection mechanisms of gut osmolality change. *Nature* 602 468–474. 10.1038/s41586-021-04359-5 35082448

[B20] Keramati M. Durand A. Girardeau P. Gutkin B. Ahmed S. H. (2017). Cocaine addiction as a homeostatic reinforcement learning disorder. *Psychol. Rev.* 124 130–153. 10.1037/rev0000046 28095003

[B21] Keramati M. Gutkin B. (2014). Homeostatic reinforcement learning for integrating reward collection and physiological stability. *eLife* 3:e04811. 10.7554/eLife.04811 25457346 PMC4270100

[B22] Lee S. Augustine V. Zhao Y. Ebisu H. Ho B. Kong D. (2019). Chemosensory modulation of neural circuits for sodium appetite. *Nature* 568 93–97. 10.1038/s41586-019-1053-2 30918407 PMC7122814

[B23] Marr D. (2010). *Vision: A Computational Investigation Into the Human Representation and Processing of Visual Information.* Cambridge, MA: MIT Press.

[B24] Matsuda T. Hiyama T. Y. Niimura F. Matsusaka T. Fukamizu A. Kobayashi K. (2017). Distinct neural mechanisms for the control of thirst and salt appetite in the subfornical organ. *Nat. Neurosci.* 20 230–241. 10.1038/nn.4463 27991901

[B25] McFarland D. (1969). Separation of satiating and rewarding consequences of drinking. *Physiol. Behav.* 4 987–989. 10.1016/0031-9384(69)90054-7

[B26] Melmed S. Auchus R. J. Goldfine A. B. Koenig R. J. Rosen C. J. (2019). *Williams Textbook of Endocrinology*, 14th Edn. Philadelphia: Elsevier, Inc.

[B27] Nakanishi S. Hikida T. Yawata S. (2014). Distinct dopaminergic control of the direct and indirect pathways in reward-based and avoidance learning behaviors. *Neuroscience* 282 49–59. 10.1016/j.neuroscience.2014.04.026 24769227

[B28] Oka Y. Butnaru M. von Buchholtz L. Ryba N. J. P. Zuker C. S. (2013). High salt recruits aversive taste pathways. *Nature* 494 472–475. 10.1038/nature11905 23407495 PMC3587117

[B29] Park S. Williams K. W. Liu C. Sohn J. W. (2020). A neural basis for tonic suppression of sodium appetite. *Nat. Neurosci.* 23 423–432. 10.1038/s41593-019-0573-2 31959933 PMC7065971

[B30] Petzschner F. H. Garfinkel S. N. Paulus M. P. Koch C. Khalsa S. S. (2021). Computational models of interoception and body regulation. *Trends Neurosci.* 44 63–76. 10.1016/j.tins.2020.09.012 33378658 PMC8109616

[B31] Pezzulo G. Rigoli F. Friston K. (2015). Active Inference, homeostatic regulation and adaptive behavioural control. *Prog. Neurobiol.* 134 17–35. 10.1016/j.pneurobio.2015.09.001 26365173 PMC4779150

[B32] Resch J. M. Fenselau H. Madara J. C. Wu C. Campbell J. N. Lyubetskaya A. (2017). Aldosterone-sensing neurons in the NTS exhibit state-dependent pacemaker activity and drive sodium appetite via synergy with angiotensin II signaling. *Neuron* 96 190.e–206.e. 10.1016/j.neuron.2017.09.014 28957668 PMC5637454

[B33] Richter C. P. (1936). Increased salt appetite in adrenalectomized rats. *Am. J. Physiol. Legacy Content* 115 155–161. 10.1152/ajplegacy.1936.115.1.155

[B34] Sánchez-Lara K. Sosa-Sánchez R. Green-Renner D. Rodríguez C. Laviano A. Motola-Kuba D. (2010). Influence of taste disorders on dietary behaviors in cancer patients under chemotherapy. *Nutr. J.* 9:15. 10.1186/1475-2891-9-15 20334666 PMC2858711

[B35] Sandhu E. C. Fernando A. B. P. Irvine E. E. Tossell K. Kokkinou M. Glegola J. (2018). Phasic stimulation of midbrain dopamine neuron activity reduces salt consumption. *eNeuro* 5:ENEURO.0064–18.2018. 10.1523/ENEURO.0064-18.2018 29766048 PMC5952649

[B36] Schultz W. (2015). Neuronal reward and decision signals: from theories to data. *Physiol. Rev.* 95 853–951. 10.1152/physrev.00023.2014 26109341 PMC4491543

[B37] Steinbach S. Hummel T. Böhner C. Berktold S. Hundt W. Kriner M. (2009). Qualitative and quantitative assessment of taste and smell changes in patients undergoing chemotherapy for breast cancer or gynecologic malignancies. *J. Clin. Oncol.* 27 1899–1905. 10.1200/JCO.2008.19.2690 19289621

[B38] Stephan K. E. Manjaly Z. M. Mathys C. D. Weber L. A. E. Paliwal S. Gard T. (2016). Allostatic self-efficacy: A metacognitive theory of dyshomeostasis-induced fatigue and depression. *Front. Hum. Neurosci.* 10:550. 10.3389/fnhum.2016.00550 27895566 PMC5108808

[B39] Sutton R. S. Barto A. G. (2018). *Reinforcement Learning: An Introduction*, 2nd Edn. Cambridge, MA: The MIT Press.

[B40] Tindell A. J. Smith K. S. Berridge K. C. Aldridge J. W. (2009). Dynamic computation of incentive salience: “wanting” what was never “liked.”. *J. Neurosci.* 29 12220–12228. 10.1523/JNEUROSCI.2499-09.2009 19793980 PMC2792765

[B41] Tindell A. J. Smith K. S. Peciña S. Berridge K. C. Aldridge J. W. (2006). Ventral pallidum firing codes hedonic reward: when a bad taste turns good. *J. Neurophysiol.* 96 2399–2409. 10.1152/jn.00576.2006 16885520

[B42] Uchida Y. Hikida T. Yamashita Y. (2021). Computational Mechanisms of Osmoregulation: A Reinforcement Learning Model for Sodium Appetite. *bioRxiv.* [Preprint]. 10.1101/2021.04.20.440596. PMC9160331 35663557

[B43] Verharen J. P. H. Roelofs T. J. M. Menting-Henry S. Luijendijk M. C. M. Vanderschuren L. J. M. J. Adan R. A. H. (2019). Limbic control over the homeostatic need for sodium. *Sci. Rep.* 9:1050. 10.1038/s41598-018-37405-w 30705296 PMC6355778

[B44] Watanabe E. Fujikawa A. Matsunaga H. Yasoshima Y. Sako N. Yamamoto T. (2000). Na v2/NaG channel is involved in control of salt-intake behavior in the CNS. *J. Neurosci.* 20 7743–7751. 11027237 10.1523/JNEUROSCI.20-20-07743.2000 PMC6772860

[B45] Zhang J. Berridge K. C. Tindell A. J. Smith K. S. Aldridge J. W. (2009). A neural computational model of incentive salience. *PLoS Comput. Biol.* 5:e1000437. 10.1371/journal.pcbi.1000437 19609350 PMC2703828

