# Supplementary material for: Computational Mechanisms of Osmoregulation: A Reinforcement Learning Model for Sodium Appetite
Source: Front Neurosci. 2022 May 19;16:857009. doi: 10.3389/fnins.2022.857009 (PMC9160331; doi:10.3389/fnins.2022.857009)
Supplement: Supplementary file 1 [file Table_1.DOCX]

Supplementary Tables

# Supplementary Table 1

**Table S1.** Parameters for the simulation experiment 1.

# Supplementary Table 2

**Table S2.** Parameters for the simulation experiment 2.

# Supplementary Table 3

# Table S3. Parameters for the simulation experiment 3.

# Supplementary Table 4

**Table S4.** Parameters for the simulation experiment 4.
